# Gene flow in Argentinian sunflowers as revealed by genotyping‐by‐sequencing data

**DOI:** 10.1111/eva.12527

**Published:** 2017-12-03

**Authors:** Ana Mondon, Gregory L. Owens, Mónica Poverene, Miguel Cantamutto, Loren H. Rieseberg

**Affiliations:** ^1^ Centro de Recursos Naturales Renovables de la Zona Semiárida (CERZOS) CCT Bahía Blanca Provincia de Buenos Aires Argentina; ^2^ Department of Botany and Biodiversity Research Centre University of British Columbia Vancouver BC Canada; ^3^ Dpto. Agronomía Universidad Nacional del Sur (UNS) Bahía Blanca Argentina; ^4^ Estación Experimental Agropecuaria Instituto Nacional de Tecnología Agropecuaria (INTA) Hilario Ascasubi Argentina

**Keywords:** crop‐wild, GMO escape, *Helianthus*, hybridization, introgression, sunflower, weeds

## Abstract

Gene flow can have several different applied consequences, ranging from extinction to the escape of transgenes to the evolution of weedy or invasive lineages. Here, we describe patterns of hybridization and gene flow involving domesticated and wild sunflowers in Argentina. To address the risks of introgression of variants from the cultivated sunflower into invasive wild *Helianthus*, we used genotyping‐by‐sequencing (GBS) to genotype 182 samples from 11 sites in Argentina, along with previously published data from samples from the native range (North America), to determine the native source populations of the Argentinian samples and to detect admixture. We unexpectedly discovered two distinctive forms of *H. petiolaris* in Argentina, one from *H. petiolaris* subsp. *petiolaris* as expected, but the other from an unknown source. Extensive admixture was observed among Argentinian sunflowers, largely confirming phenotypic predictions. While many hybrids are F1s, there were signals consistent with introgression from the domesticated sunflower into *H. petiolaris*. Whether this introgression is incidental or a causal driver of invasiveness is not yet clear, but it seems likely that genes found in the domesticated sunflower genome (whether engineered or not) will quickly find their way into wild Argentinian sunflower populations.

## 1 INTRODUCTION

A recent special issue in this journal (Ellstrand & Rieseberg, 2016) highlighted the potentially important applied consequences of gene flow. These included genetic rescue (Fitzpatrick et al., 2016), demographic or genetic swamping (Todesco et al., 2016), transgene escape (Lu et al., 2016), and the evolution of weedy or invasive taxa (Welles & Ellstrand, 2016). Gene flow between wild and domesticated species can be especially problematic because of the likely imbalance in population size between the crop and nearby wild relative populations and because some domestication traits also contribute weedy life‐history behaviors (Ellstrand et al., 2013).

In this article, we focus on gene flow involving the domesticated sunflower, *Helianthus annuus* L. var *macrocarpus* (DC.), its wild progenitor, *H. annuus,* and a compatible wild relative, *H. petiolaris* Nutt. in Argentina. Such gene flow has been associated with the formation of aggressive weedy sunflowers in Europe (Muller, Latreille, & Tollon, 2011), Israel, and Australia (Lai et al., 2012). Understanding gene flow in Argentinian sunflowers is critical because Argentina is both a large domestic sunflower producer and has introduced weedy sunflowers, heightening the risk of GMO escape. Whether domestic alleles can introgress into both weedy species or just the wild progenitor, *H. annuus*, has important implications in how crop‐wild gene flow should be managed.

Sunflower is one of the world's most important oilseed crops (www.faostat.fao.org), with Argentina ranking as the fourth largest producer (www.agroindustria.gob.ar). In contrast to most other leading oilseeds, however, genetically engineered sunflower cultivars have not been commercialized because of a combination of economic and ecological considerations (Cantamutto & Poverene, 2007, 2010). From an economic standpoint, the main worry was that the release of GM sunflower cultivars would harm the marketing of sunflower oil (Cantamutto & Poverene, 2007), whereas ecological concerns stemmed from the possibility that transgenes would spread into compatible wild and weedy sunflower species (Snow et al., 2003).

While industry largely discontinued its sunflower transformation programs in the late 1990s or early 2000s, interest has been re‐awakened due to the development of facile genome editing approaches, such as CRISPR/Cas9 (Deltcheva et al., 2011; Jinek et al., 2012). These new approaches are much more precise than classic transgenic methods and do not require the introduction of foreign genetic material (or such material can be removed prior to commercialization). As a consequence, regulatory hurdles are likely to be lower and public reaction less hostile (National Research Council of the National Academies, 2016), rekindling interest in crop‐wild gene flow and its consequences in sunflower and other crops.

*Helianthus annuus* and *H. petiolaris,* the species targeted in this study, are native to the great plains of North America, as well as to parts of the southwestern USA. However, in recent years, a ruderal form of *H. annuus* has been reported in several other countries, including Australia (Seiler, Gulya, Kong, Thompson, & Mitchell, 2008) and Argentina (Poverene, Cantamutto, & Seiler, 2008). *H. petiolaris* appears to be less invasive, but it has colonized Argentina as first reported by Covas (1966) in La Pampa province. Although the presence of *H. annuus* was also reported around this time (Cabrera, 1974; Covas, 1964), the morphology did not match that of wild or weedy *H. annuus*, but rather escaped domestic sunflower. It was another 40 years before wild *H. annuus* was described in Argentina (Poverene et al., 2002). *Helianthus petiolaris* is thought to have been introduced to Argentina as a forage seed contaminant, while accessions of *H. annuus* likely were imported as forage plants and/or sunflower germplasm. In both cases, there are no written reports of the introduction (A. Luciano, pers comm). Wild *H. annuus* has been occasionally used in breeding programs since the late 1940s (Bauer, 1988; Bertero de Romano & Vázquez, 2003), but escapes from breeding stations were deemed unlikely by Cantamutto, Torres, et al. (2010) because ruderal populations or herbarium specimens of wild *H. annuus* have not been found nearby. Both species currently occur as far as 500 km from the presumptive site of introduction in the center of the country. Their rapid post introduction dispersal has likely been aided by vehicles and farm machinery (Cantamutto, Torres, et al., 2010).

The two species have different soil preferences; *H. petiolaris* occurs predominantly in sandy soils, while *H. annuus* prefers heavier, loamy soils (Cantamutto, Poverene, & Peinemann, 2008; Heiser, 1947). Although distinct, these two kinds of soil types often co‐occur in agricultural ecosystems (Cantamutto, Torres, et al., 2010). As a consequence, the two species are frequently found in sympatry, with a patchy distribution, usually on the roadsides of disturbed agroecosystems, and often in contact with cultivated sunflower (Poverene et al., 2008). Over the years, off‐type individuals that exhibit intermediate morphological traits between the two species—and might be the product of hybridization—have been reported at these sites (Ureta, Cantamutto, Carrera, Delucchi, & Poverene, 2008).

In North America, hybrid zones between *H. annuus* and *H. petiolaris* are frequently reported in disturbed environments in central and western USA (Heiser, 1947; Kane et al., 2009; Yatabe, Kane, Scotti‐Saintagne, & Rieseberg, 2007). Gene flow is bidirectional, and frequencies of introgression decrease toward the edges of the zones (Rieseberg, Whitton, & Gardner, 1999). In addition, three *bona fide* hybrid species are known to have arisen from hybrid zones of the two species (Rieseberg, 1991). Thus, it would not be surprising if hybridization is occurring in Argentina as well.

In addition to the possibility of hybridization involving wild *Helianthus* species, off‐type plants that are thought to be hybrids between *H. petiolaris* and the domestic sunflower have been described in Argentina in areas where the two species overlap (Covas & Vargas López, 1970; Ferreira, 1980). Hybridization between the domestic sunflower and its wild relatives has been studied in detail in North America, driven in part by concerns about the possibility of transgene escape (Arias & Rieseberg, 1994; Burke & Rieseberg, 2003; Rieseberg, Kim, & Seiler, 1999). First generation hybrids between cultivated *H. annuus* and *H. petiolaris* can be readily produced by artificial crosses, but are highly sterile suggesting that even the presence of hybrids does not necessarily indicate effective gene flow (Ungerer, Baird, Pan, & Rieseberg, 1998). In the invaded environments, first‐generation hybrids between domestic sunflower and *H. petiolaris* are frequent; however, they are partially sterile and it is unknown if effective introgression occurs (Gutierrez et al., 2010; Ureta et al., 2008).

In light of extensive and well‐documented hybridization and introgression between *Helianthus* species in North America, and phenotypic reports suggestive of hybridization in Argentina, we employed a genotyping‐by‐sequencing (GBS) approach to address the following two questions: (i) Are the wild species currently hybridizing in Argentina and does this account for observations of so‐called off‐type individuals in areas of sympatry? and (ii) Are domesticated sunflower alleles introgressing into wild Argentinian populations? Our results suggest that crop‐wild gene flow is ongoing and highlight the future risk that edited sunflower genes will escape from farmer's field.

## 2 MATERIALS AND METHODS

### 2.1 Study sites and Sample collection

Five areas in Argentina where off‐type *Helianthus* individuals are frequently observed were surveyed (Table 1): Catriló (CAT), Colonia Barón (BAR) and Winifreda (WIN), in La Pampa province; Carhué (CHU) and Trenque Lauquen (CZ), both from Buenos Aires province. They were located along the sides of dirt roads and next to fields often cultivated with sunflower. It is noteworthy that all of these sites are subject to frequent disturbance from agricultural and road machinery. Plant populations along roads spanned about 3‐10 m wide and ranged from 100 m to more than 1 km long. In three sites (BAR, CHU, and WIN), plants corresponding to the *Helianthus petiolaris* and *H. annuus* biotypes were identified. In CAT and CZ, no biotypes of ruderal *H. annuus* were recorded, but the domesticated sunflower, both cultivated and escaped, were common. Plants were classified a priori into three biotypes, according to their morphology: *H. annuus* (herein ANN), *H. petiolaris* (PET), and off‐type (OT), which matches neither species, following Ureta et al. (2008). Leaf samples were stored at ‐80°C, lyophilized, and ground with mortar and pestle.

**Table 1:** Sampled populations, geographic origin, and putative biotypes present

Population	Biotype	Nearby locality	Code	Latitude	Longitude	Samples	Sympatry	Crop presence
*Helianthus annuus* ^1^	ANN	Diamante	DIA	−32.0603	−60.6453	5	No	No
*H. annuus* ^1^	ANN	Río Cuarto	RCU	−33.1603	−64.3358	5	No	No
*H. petiolaris* ^1^	PET	Hilario Lagos	HIL	−34.9489	−63.9283	2	No	No
*H. petiolaris* ^1^	PET	Saliquelló	SAL	−36.8097	−62.9917	2	No	Yes
*H. petiolaris* ^1^	PET	Unión	UNI	−35.1353	−65.9369	2	No	Yes
*H. petiolaris* ^1^	PET	Santa Rosa	SAN	−36.31	−64.2836	2	No	No
Both	PET, ANN, OT	Winifreda	WIN	−36.1753	−64.2053	12, 10, 1	Yes	Yes
Both	PET, ANN, OT	Carhué	CHU	−37.2414	−62.8131	16, 17, 12	Yes	Yes
Both	PET, ANN, OT	Colonia Barón	BAR	−36.0044	−63.8297	14, 16, 18	Yes	Yes
*H. petiolaris*	PET, ANN, OT	Catriló	CAT	−36.435	−63.4369	15, 11, 4	No	Yes
*H. petiolaris*	PET, OT	Trenque Lauquen	CZ	−35.8222	−62.7669	9, 9	No	Yes

^1^Collected and described in Poverene et al. (2008).

Tissue samples from *H. annuus* and *H. petiolaris* biotypes from previously surveyed populations that show no phenotypic evidence of admixture were also genotyped. Additionally, we genotyped a single hand‐crossed F1 individual whose parents were a male‐sterile domestic *H. annuus* and a wild *H. petiolaris* (Table 1).

### 2.2 DNA extraction, library preparation, and sequencing

DNA extraction was carried out using a CTAB protocol (CIMMYT, 2005), starting from 10 mg of dried tissue. After DNA quantification with a Qubit 2.0 Fluorometer (Thermo Fisher Scientific), two GBS libraries (96 samples each) were developed following Elshire et al. (2011), with minor modifications as described in Owens, Baute, and Rieseberg (2016). Each library was sequenced on a single lane of the Illumina HiSeq2000 with 100‐bp paired‐end reads, at the UBC Biodiversity Research Center sequencing facility in Vancouver, Canada.

### 2.3 SNP calling

In addition to the GBS data described above, we employed a set of previously described RNAseq samples from North American native wild and domesticated *Helianthus annuus* (Renaut & Rieseberg, 2015) to aid with analyses of introgression involving the domesticated sunflower. Also, GBS samples of *H. maximiliani*,* H. petiolaris*,* H. debilis*,* H. praecox,* and *H. niveus* (Baute, Owens, Bock, & Rieseberg, 2016) from the native range were incorporated as a reference to help to elucidate the native source populations of ruderal Argentinian *Helianthus* (Table S1).

The new GBS reads were demultiplexed using an in‐house Perl script that also trims off adapter read‐through (Owens et al., 2016). Reads shorter than 50 bp following this trimming step were removed. The remaining reads were aligned to a genome assembly of *H. annuus* (v1.1.bronze; http://www.sunflowergenome.org) using “NextGenMap” (Sedlazeck, Rescheneder, & von Haeseler, 2013) for GBS samples or “BWA” and “subjunc” for RNAseq sequence data (Li & Durbin, 2010; Liao, Smyth, & Shi, 2013). Alignments were converted to binary format using “SAMtools” (version: 0.1.19) (Li et al., 2009). Read group information and PCR duplicate marking were completed using “Picard” (version: 1.114) (http://broadinstitute.github.io/picard). Genotyping was performed using the “HaplotypeCaller” and “GenotypeGVCFs” commands in GATK (version: 3.3) (Van der Auwera et al., 2002) together in series. All scripts used can be found on Github (https://github.com/owensgl/argentina_helianthus), and all raw demultiplexed data were deposited in the SRA (PRJNA359995). For each dataset, we filtered for genotypes with ≥5 reads and biallelic sites with >80% sample coverage and >5% minor allele frequency. The number of SNPs per dataset is reported in Table S2. We did not apply a maximum read depth filter because our GBS protocol produces highly variable read depth. Despite this, only 0.1% of sites showed observed heterozygosity above 60%, suggesting that paralogs were not a large issue in this dataset.

### 2.4 Data analysis

Collections in Argentina included samples identified as *H. annuus* (ANN), *H. petiolaris* (PET), and intermediate plants (OT). To confirm sample identification and classify intermediate plants, we ran a structure analysis using NGSadmix and fastSTRUCTURE (Raj, Stephens, & Pritchard, 2014; Skotte, Korneliussen, & Albrechtsen, 2013). Both programs identify admixture proportions but NGSadmix uses genotype likelihoods, whereas fastSTRUCTURE uses SNP calls. For both methods, we chose 2–6 groups by specifying the K parameter.

To determine whether OT samples were F1 or later generation hybrids, we used the R program HIest to estimate admixture proportion and interspecific heterozygosity (Fitzpatrick, 2012). In this analysis, we treated allopatric single species populations as pure types; *H. annuus* (samples RCU and DIA) and *H. petiolaris* (samples HIL, SAL, SAN, and UNI, see Table 1). As stochastic variation at low read depth can cause heterozygote dropout, for this analysis we only used genotypes with ≥10 reads and filtered our set to contain only sites where there was a fixed difference between the pure groups. Also genotyped with our study samples is one known F1 between a male‐sterile (CMS‐PET1) domestic *H. annuus* and *H. petiolaris*. This sample was used as a control.

To explore the genetic diversity in Argentinian wild sunflowers, we used a principal component analysis in the R program SNPrelate (Zheng, Levine, Gogarten, Laurie, & Weir, 2012). Sites were filtered for linkage (LD < 0.2) using the function *snpgdsLDpruning*. We also tried more (LD < 0.05) and less (LD < 0.4) stringent linkage filtering and found the overall pattern unchanged. Bimodal genetic structure in *H. petiolaris* may be due to presence of both subspecies of *H. petiolaris* in Argentina or represents the presence of genetic ancestry from a third *Helianthus* species. To test this hypothesis, we included sequenced samples from all species within the petiolaris clade of *Helianthus*:* H. petiolaris* ssp. *petiolaris*,* H. petiolaris* ssp. *fallax*,* H. debilis*,* H. praecox,* and *H. niveus*. We reran the principal component analysis with each new taxon to see whether it clustered with one of the Argentinian *H. petiolaris* groups. To further visualize the genetic relationships, we ran SplitsTree4 to create a phylogenetic network of all Argentinian and North American *Helianthus* samples. Lastly, we calculated Weir and Cockerham's F_ST_ between genetically pure *H. annuus* and pure *H. petiolaris* (as identified by NGSadmix), as well as between the two subgroups of *H. petiolaris* (Weir & Cockerham, 1984). As a reference, we also calculated F_ST_ between the North American species and subspecies listed above. F_ST_ was calculated using a custom perl script and required a minor allele frequency of ≥5% and at least three individuals genotyped in each population. The minimum individual value was picked due to limitations in sample size for some species.

Besides the possibility of *H. annuus* x *H. petiolaris* hybridization, the Argentinian populations were located in the same region as cultivated sunflower, and they were likely subjected to the crop pollen flow. To assess this, we used a set of RNAseq samples from domesticated and wild native *H. annuus* (see above) and calculated Patterson's D‐statistic using the multipop abbababa2 function in ANGSD (Korneliussen, Albrechtsen, & Nielsen, 2014; Patterson et al., 2012). This test uses a four‐member phylogeny and asks whether derived alleles are shared between two members of the phylogeny more than you would expect based on their positions in the tree. In our study, we used two different strategies. First, we tested whether each population of pure *H. annuus* was closer to domestic *H. annuus* than to a set of wild native North American *H. annuus* (Figure 5a). This asks whether there is contemporary gene flow between Argentinian *H. annuus* and domestic *H. annuus*. Secondly, we asked whether native wild *H. annuus* or domestic *H. annuus* was closer to Argentinian *H. petiolaris* (Figure 5b). This will show whether domestic *H. annuus* alleles have introgressed into Argentinian *H. petiolaris*. As a reference, we also tested North American *H. petiolaris* samples for introgression in the same scenario. We included samples of *H. maximiliani*, a diploid perennial sunflower to act as an outgroup to both *H. annuus* and *H. petiolaris*.

## 3 RESULTS

Analyses included 64 individuals that were morphologically identified as *Helianthus annuus*, 74 individuals identified as *H. petiolaris*, and 44 off‐type individuals (Table 1). Initial variant calling produced 112,267 variants, including indels and SNPs. After removing indels and filtering for genotype depth, sample coverage and minor allele frequency, 3,526 SNPs remained for analysis. Read depth of SNPs used is plotted in Fig. S1. Due to the GBS method employed, we did not genotype any SNPs on the cytoplasmic genomes; thus, we restrict our analysis to only the nuclear genome.

**Figure 1:**
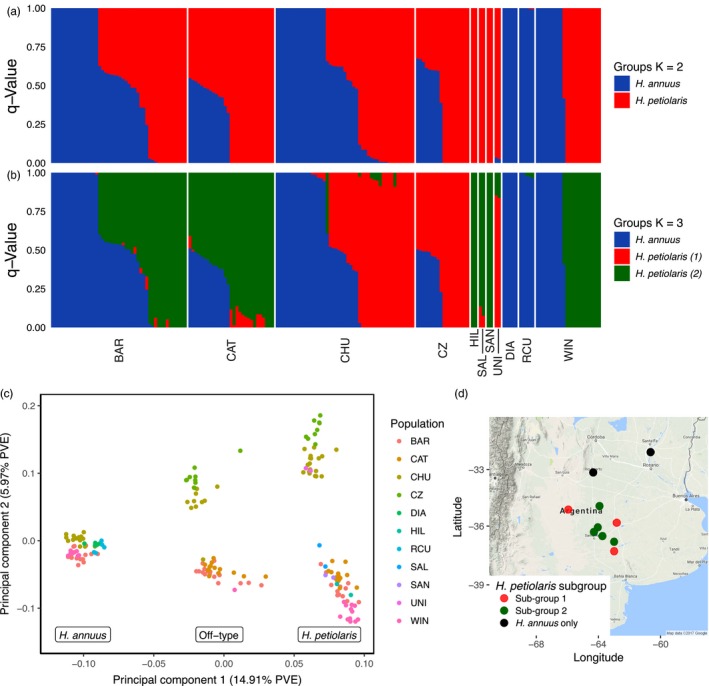
Structure of wild *Helianthus* populations in Argentina at K = 2 (a) and K = 3 (b). Each vertical line represents an individual, and different colors indicate its proportion of membership to the inferred gene pools. Sites are arranged according to Table 1. (c) Principal component analysis of GBS data from *H. annuus*,* H. petiolaris*, and off‐types from 11 Argentinian localities. (d) Geographic location of the two *H. petiolaris* genetic subgroups

Structure analyses performed with NGSadmix and fastSTRUCTURE delivered consistent results (Figure 1a). FastStructure selected two as the best K value, while NGSadmix does not pick an optimal value. We focus on K = 2 and K = 3 because they best show the major structure of the populations. When run at K = 2, NGSadmix showed a correspondence between the genomic composition of the samples and their a priori classification, based on morphological characters (90% agreement). This meant that individuals with a typical *H. annuus* morphology clustered together and those with an *H. petiolaris*‐like morphology did the same. Most of the morphologically intermediate plants showed evidence of both clusters in their genome and were classified as hybrids. Surprisingly, all *H. annuus*‐like plants from CAT possessed a hybrid genomic composition; in that location, we found no pure wild *H. annuus* samples. In all populations containing both *H. annuus*‐like and *H. petiolaris*‐like individuals, hybrids were detected, suggesting hybridization is common when the species are in sympatry.

At K = 3 (Figure 1b), *H. annuus*‐like plants remained assigned to a single cluster (blue in Figure 1), but *H. petiolaris*‐like plants were split into two groups: one of the clusters included samples from Trenque Lauquen, Carhué, and Unión (red in Figure 1), and the other one included plants from Catriló, Colonia Barón, Winifreda, Hilario Lagos, Saliquelló, and Santa Rosa (green in Figure 1). We detected low levels of admixture between the two *H. petiolaris* subgroups in some samples, although all *H. petiolaris* populations had a majority of their ancestry from one of the two subgroups.

**Figure 2:**
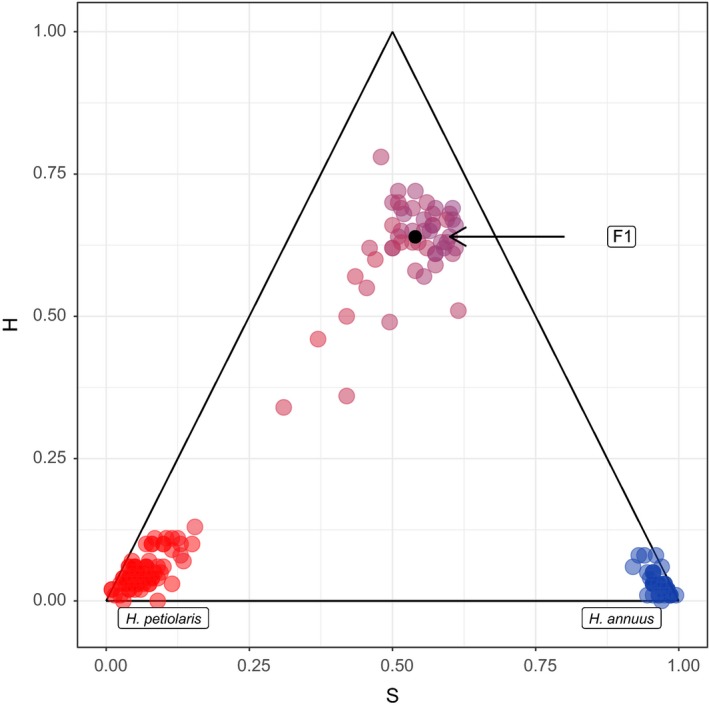
Distribution of ancestry (S) and heterozygosity (H) in Argentinian *Helianthus* samples, based on HIest analysis. F1 corresponds to an artificial crop (*H. annuus*) x *H. petiolaris* cross. Each sample is color coded based on the NGSadmix analysis (K = 2)

As hybrid zones typically exhibit a continuum of hybrid classes, individuals were classified by estimates of both ancestry (S) and interclass heterozygosity (H) according to Fitzpatrick (2012) using the R package Hlest. This analysis (Figure 2) found that most morphologically intermediate individuals had an interspecific heterozygosity of approximately 0.65, lower than the expected value for F1s (1.0) but above that for F2s (0.5). The known F1 sample had an interspecific heterozygosity of 0.64, suggesting that most of the samples are in fact first generation hybrids, but that interspecific heterozygosity is lower than expected. This might be due to the species‐specific SNPs identified from allopatric populations having polymorphism in hybridizing populations and/or because of reduced power to detect heterozygotes with GBS data.

Results from the PCA of Argentinian populations confirmed the NGSadmix results. The first component (14.91% variation explained) differentiated *H. annuus* from *H. petiolaris* and placed morphologically intermediate samples in the middle (Figure 1c). In the second component (5.97% variation explained), the *H. annuus* samples remained as a single well‐defined cluster, but the *H. petiolaris* and intermediate plants were split into two groups, which corresponded to those found in the NGSadmix analysis with three groups (K = 3 in Figure 1b). More generally, individuals from the same species and population clustered close to each other in the PCA.

**Figure 3:**
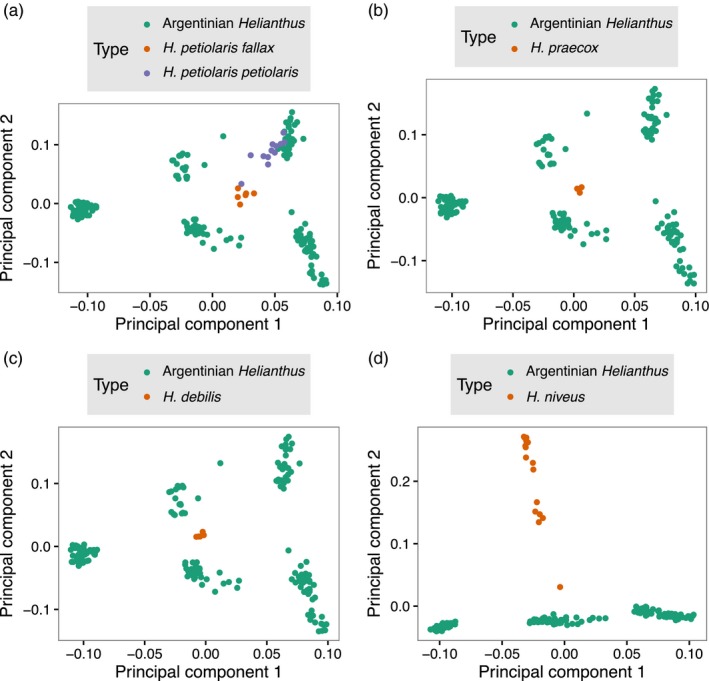
Principal component analysis of GBS data from US and Argentinian samples of *Helianthus*. Individual graphs include a) *H. petiolaris fallax* and *H. petiolaris petiolaris* b) *H. praecox*, c) *H. debilis* and d) *H. niveus*.

**Figure 4:**
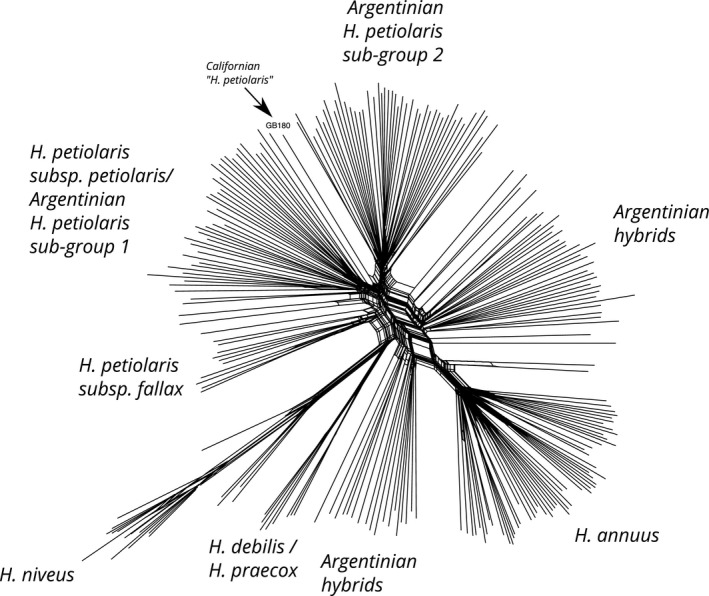
Split network analysis of Argentinian and North American *Helianthus* GBS samples. Sample GB180, which is most closely related to the unknown Argentinian *H. petiolaris* subgroup 2, is highlighted

Interestingly, the two different *H. petiolaris* genomic subgroups identified by PCA (Figure 1c) were found to be separable geographically as well, with one subgroup corresponding to the geographic center of the studied region, and the other found more at the edges of this region (Figure 1d). In further PCAs, only *H. petiolaris* spp *petiolaris* clustered with one of the Argentinian subgroups, whereas the remaining Argentinian materials showed no affinity with North American species (Figure 3). Interestingly, in the SplitsTree4 analysis, the non‐Argentinian sample that is most closely related to unknown *H. petiolaris* subgroup is, itself, of uncertain origin (Figure 4). This sample, labeled GB180 from accession PI 468788, was collected in central California, USA, and is classified as *H. niveus subsp, canescens* but is genetically closer to *H. petiolaris* (Baute et al., 2016). We found moderate genetic divergence between the two *H. petiolaris* subgroups (F_ST_ = 0.198), slightly higher than the divergence between *H. petiolaris subsp. petiolaris* and *H. petiolaris subsp. fallax* (F_ST_ = 0.151), but less than between species (F_ST_ = 0.3–0.4) (Table S3).

**Figure 5:**
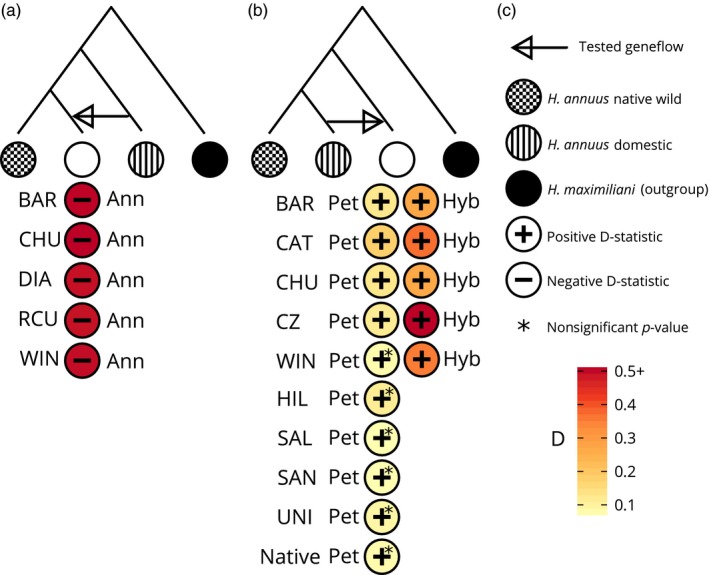
ABBA‐BABA or D‐statistic tests for gene flow from domestic *Helianthus annuus*. (a) Testing gene flow from domestic *H. annuus* into each Argentinian *H. annuus* population. (b) Testing gene flow from domestic *H. annuus* into each Argentinian *H. petiolaris* populations. Samples are divided into pure (Pet) or admixed (Hyb) based on NGSadmix results. Native *H. petiolaris* samples are from North America. (c) Legend for symbols used. *p*‐values were corrected for multiple testing using Bonferroni correction. Corrected *p*‐value <.05 was considered significant

Hybrids may be the product of wild‐to‐wild hybridization or the result of pollen flow from domesticated *H. annuus* also growing in the area. Considering the overlap of most of the Argentinian *Helianthus* populations with the production area of cultivated sunflower, we estimated Patterson's D‐statistic to determine whether domestic *H. annuus* is introgressing alleles into Argentinian *Helianthus* (Table 2 and Table S4). In the first test, we found a consistent significantly negative signal for all populations, suggesting greater sharing of derived alleles between domestic *H. annuus* and wild North American *H. annuus*, rather than Argentinian *H. annuus* (Figure 5a). For the second test, we found a consistent positive signal suggesting greater derived allele sharing between Argentinian *H. petiolaris* and domestic *H. annuus*, rather than wild North American *H. annuus* (Figure 5b). Although all populations showed a positive signal, not all were significant after correcting for multiple testing. Importantly, North American *H. petiolaris* also produced a positive signal, but was not significant after multiple testing corrections and was less than most of the Argentinian populations.

**Table 2:** Results of ABBA‐BABA (D‐statistic) tests

Tested Species	D	*p*‐value	Bonferroni *p*‐value	H1	H2	H3	H4
*Helianthus annuus*	−0.766702	0	0	Domestic *H. annuus*	BAR	Wild NA H. annuus	*H. maximiliani*
*H. annuus*	−0.770567	0	0	Domestic *H. annuus*	CHU	Wild NA H. annuus	*H. maximiliani*
*H. annuus*	−0.747214	0	0	Domestic *H. annuus*	DIA	Wild NA H. annuus	*H. maximiliani*
*H. annuus*	−0.744409	0	0	Domestic *H. annuus*	RCU	Wild NA H. annuus	*H. maximiliani*
*H. annuus*	−0.752907	0	0	Domestic *H. annuus*	WIN	Wild NA H. annuus	*H. maximiliani*
*H. petiolaris*	0.273886	0	0	Wild NA *H. annuus*	Domestic *H. annuus*	BAR Hybrids	*H. maximiliani*
*H. petiolaris*	0.126935	.001319	.019785	Wild NA *H. annuus*	Domestic *H. annuus*	BAR *H. petiolaris*	*H. maximiliani*
*H. petiolaris*	0.369036	0	0	Wild NA *H. annuus*	Domestic *H. annuus*	CAT Hybrids	*H. maximiliani*
*H. petiolaris*	0.180477	.000002	.00003	Wild NA *H. annuus*	Domestic *H. annuus*	CAT *H. petiolaris*	*H. maximiliani*
*H. petiolaris*	0.270556	0	0	Wild NA *H. annuus*	Domestic *H. annuus*	CHU Hybrids	*H. maximiliani*
*H. petiolaris*	0.12798	.000481	.007215	Wild NA *H. annuus*	Domestic *H. annuus*	CHU *H. petiolaris*	*H. maximiliani*
*H. petiolaris*	0.505836	0	0	Wild NA *H. annuus*	Domestic *H. annuus*	CZ Hybrids	*H. maximiliani*
*H. petiolaris*	0.119033	.00209	.03135	Wild NA *H. annuus*	Domestic *H. annuus*	CZ *H. petiolaris*	*H. maximiliani*
*H. petiolaris*	0.114042	.023592	.35388	Wild NA *H. annuus*	Domestic *H. annuus*	HIL *H. petiolaris*	*H. maximiliani*
*H. petiolaris*	0.073794	.110138	1	Wild NA *H. annuus*	Domestic *H. annuus*	SAL *H. petiolaris*	*H. maximiliani*
*H. petiolaris*	0.073788	.125875	1	Wild NA *H. annuus*	Domestic *H. annuus*	SAN *H. petiolaris*	*H. maximiliani*
*H. petiolaris*	0.092248	.043346	.65019	Wild NA *H. annuus*	Domestic *H. annuus*	UNI *H. petiolaris*	*H. maximiliani*
*H. petiolaris*	0.354193	0	0	Wild NA *H. annuus*	Domestic *H. annuus*	WIN Hybrids	*H. maximiliani*
*H. petiolaris*	0.079686	.064328	.96492	Wild NA *H. annuus*	Domestic *H. annuus*	WIN *H. petiolaris*	*H. maximiliani*
*H. petiolaris*	0.104493	.004149	.062235	Wild NA *H. annuus*	Domestic *H. annuus*	North American *H. petiolaris*	*H. maximiliani*

## 4 DISCUSSION

### 4.1 Origins and admixture of wild sunflowers in Argentina

Using GBS data, we have shown that Argentina is home to wild *Helianthus annuus*, wild *H. petiolaris,* and hybrids between the two species. Previous studies have identified off‐type individuals whose morphology matched neither pure species (Gutierrez et al., 2010; Ureta et al., 2008). Here, we show, using interspecific heterozygosity and ancestry proportions, that these individuals are F1 hybrids (Figures 1 and 2). In support of this conclusion, we found no off‐type or genetically hybrid individuals in allopatric populations where hybridization is not possible.

We identify hybrids between *H. annuus* and *H. petiolaris*, but is there effective gene flow between the species? Previous work has shown that strong prezygotic barriers to gene flow between *H. annuus* and *H. petiolaris*, including ecogeographic, reproductive asymmetry and pollen competition (Sambatti, Strasburg, Ortiz‐Barrientos, Baack, & Rieseberg, 2012). The fact that F1 hybrids are common may reflect a breakdown of these barriers in the invasive range or simply the numeric advantage of domestic sunflowers in a cultivated context. In addition to the prezygotic barriers, several strong postzygotic barriers exist including highly reduced F1 pollen viability and seed set. In our dataset, most hybrids are F1s but several samples where ancestry is not evenly split may represent backcrosses toward *H. petiolaris* (Figure 1a). This confirms the reproductive barriers between *H. annuus* and *H. petiolaris* do not prevent hybridization, and introgression is possible.

In addition to hybridization, we also detected two distinct population subgroupings within *H. petiolaris* in both the principal component and NGSadmix analyses (K = 3). This division is not due to varying levels of hybridization; both subgroups were equally related to *H. annuus*. The fact that subgroup assignment was largely bimodal in the PCA and considering the geographic arrangement of populations, we think it is unlikely for these subgroupings to have arisen purely from isolation by distance or drift post introduction. These subgroups are as diverged as two *H. petiolaris* subspecies (*subsp. petiolaris* and *subsp. fallax*), supporting an older origin of the clades. Convergent local adaptation is unlikely to explain the pattern; we failed to find phenotypic, soil, or climate differences between the two genetic subgroups (Cantamutto, Torres, et al., 2010); and the time frame (~50 years) is extremely short for adaptive change of this magnitude. *Helianthus petiolaris* is thought to have been introduced to Argentina as a contaminant of sorghum forage seeds imported from Texas (Cantamutto, Torres, et al., 2010), where *H. petiolaris* spp *petiolaris* is native. However, our results imply that there may have been a second introduction from an as yet unknown form of *H. petiolaris*. Based on a single poorly identified accession, this introduction may have occurred from a western USA population of *H. petiolaris*, but as our dataset does not contain any other western USA *H. petiolaris,* we cannot confirm this hypothesis (Figure 4). Future studies using wider samplings of native *H. petiolaris* will be able to better identify the source of the second introduction.

Popular structure analysis methods usually focus on revealing the degree of admixture present in populations (Falush, Stephens, & Pritchard, 2003; Pritchard, Stephens, & Donelly, 2000) but not the genealogy of the hybrids (Fitzpatrick, 2012; Gompert & Buerkle, 2016). However, in our study, interspecific heterozygosity in the artificial F1 hybrid (H = 0.65) is lower than expected, possibly because of polymorphism for putatively diagnostic SNPs in the parental populations or because of the reduced power of GBS for calling heterozygotes. That the bulk of the hybrids have a similar level of heterozygosity to the artificial F1 implies that most hybrids are F1s, but a handful of genotypes appear to represent backcrosses toward the more abundant parent, *H. petiolaris*. Our work suggests that caution should be used when identifying hybrid classes using SNP markers and that reference samples are helpful for overcoming data limitations.

### 4.2 Hybridization with the cultivated sunflower

Wild *Helianthus* species and cultivated sunflower overlap in flowering period and share the same pollinators, which results in gene flow among them in North America (Arias & Rieseberg, 1994; Burke, Gardner, & Rieseberg, 2002; Linder, Taha, Seiler, Snow, & Rieseberg, 1998; Snow, Moran‐Palma, Rieseberg, Wszelaki, & Seiler, 1998) and in Argentina (Gutierrez et al., 2010; Ureta et al., 2008) where the cultivated area overlaps with that of the wild species. Even with low introgression rates, the extent of the contact area and the number of plants involved make them worthy of attention (Poverene et al., 2008; Presotto et al., 2011).

In the present study, Patterson's D showed evidence for domestic introgression in Argentinian *H. petiolaris* but not *H. annuus* (Figure 5a). This is a surprising result considering the reduced reproductive barriers between wild and domestic *H. annuus*, but may actually be because of confounding factors in the test (Sambatti et al., 2012). The D‐statistic is an explicitly relative test; it looks for greater derived allele sharing in comparison with a reference population. Consequently, other introgression events not explicitly tested for can produce false patterns. In our case, strong introgression between North American *H. annuus* and domestic *H. annuus* could override a lesser amount of introgression between Argentinian *H. annuus* and domestic sunflower (Burke et al., 2002). Similarly, *H. petiolaris* introgression into Argentinian *H. annuus,* a likely scenario considering the level of hybridization observed here, would also produce the negative signal seen. Altogether, testing for introgression between extremely closely related populations is challenging, especially in this context, and further work is needed to quantify domestic introgression into *H. annuus*.

When testing *H. petiolaris*, we see a consistent positive D signal in all populations tested, both in hybrid and pure samples (Figure 5b). Interestingly, D was not significantly positive in all four populations where off‐type or hybrid individuals were not found, suggesting that introgression may be higher in populations where F1 hybrids are currently being produced. Importantly, D was higher in hybrids compared to pure *H. petiolaris* individuals, suggesting greater domestic ancestry in hybrids (Figure 5b). This suggests that hybrids are either produced directly from domestic *H. annuus* or from wild *H. annuus* harboring domestic introgressions. Although our result follows predictions, it is important to consider possible confounding factors. Introgression from *H. petiolaris* into domestic *H. annuus* during breeding, which occurs at a low but detectable level, could produce positive D in this scenario (Baute, Kane, Grassa, Lai, & Rieseberg, 2015). This caveat is bolstered by the fact that North American *H. petiolaris* also has a positive D score, suggesting introgression, albeit at a lower level than most of the Argentinian populations. Thus, at least some of the positive D score is likely from factors other than domestic introgression into Argentinian *H. petiolaris*. All together, our results are consistent with some gene flow from domestic *H. annuus* into *H. petiolaris*.

### 4.3 Extension of previous studies

Hybridization in wild and domestic Argentinian sunflowers has been previously studied, but here we advance our understanding in several key ways. First, we conclusively identify that off‐type individuals are largely F1 hybrids, not advanced generation hybrids. This could not be proven with previous, less dense molecular markers and is important for understanding the composition of the hybrid zones. Secondly, we show that domestic alleles are making their way into *H. petiolaris*, even in individuals where *H. annuus* ancestry is not obvious in structure results. This suggests that introgression may be subtle for *H. petiolaris* and that only looking for hybrids may underestimate the possibility of adaptive introgression from a domestic source.

## 5 CONCLUSIONS

In conclusion, the population genomic analyses reported here confirm widespread admixture between introduced wild sunflowers in Argentina. While most of the hybrids are F1s, there does appear to be successful introgression into the more abundant species, *Helianthus petiolaris,* including from domestic *H. annuus*. It is not clear whether the introgression is adaptive, as introgressed alleles may be surfing to high frequency as a result of rapid expansion of the invading ruderal sunflowers (Currat, Ruedi, Petit, & Excoffier, 2008). Nonetheless, it is clear that if genome editing begins to contribute to sunflower improvement, as seems likely, then edited genes are likely to quickly move into wild Argentinian sunflowers.

Hybridization has often been linked to the evolution of invasiveness (Ellstrand & Schierenbeck, 2000; Hovick & Whitney, 2014) and appears to be associated with the evolution of weedy sunflowers away from its center of origin (Casquero, Presotto, & Cantamutto, 2013; Lai et al., 2012; Muller et al., 2011). Thus, an important unanswered question is whether the hybridization reported here for Argentinian sunflowers is incidental or whether it is a causal driver of invasiveness.

## AUTHOR CONTRIBUTION

MP and MC localized the studied populations; MC was in charge of the handmade crosses; AM, MP, and LHR conceived of study; AM collected samples and created sequencing libraries; GLO conducted bioinformatic analyses; AM, MP, GLO, and LHR wrote the article; all authors edited and approved manuscript.

## DATA ARCHIVING STATEMENT

All sequence data are archived in the Sequence Read Archive: BioProject PRJNA359995.
