# Supplementary figures and images for: Gene flow in Argentinian sunflowers as revealed by genotyping‐by‐sequencing data
Source: Evol Appl. 2017 Dec 3;11(2):193–204. doi: 10.1111/eva.12527 (PMC5775495; doi:10.1111/eva.12527)

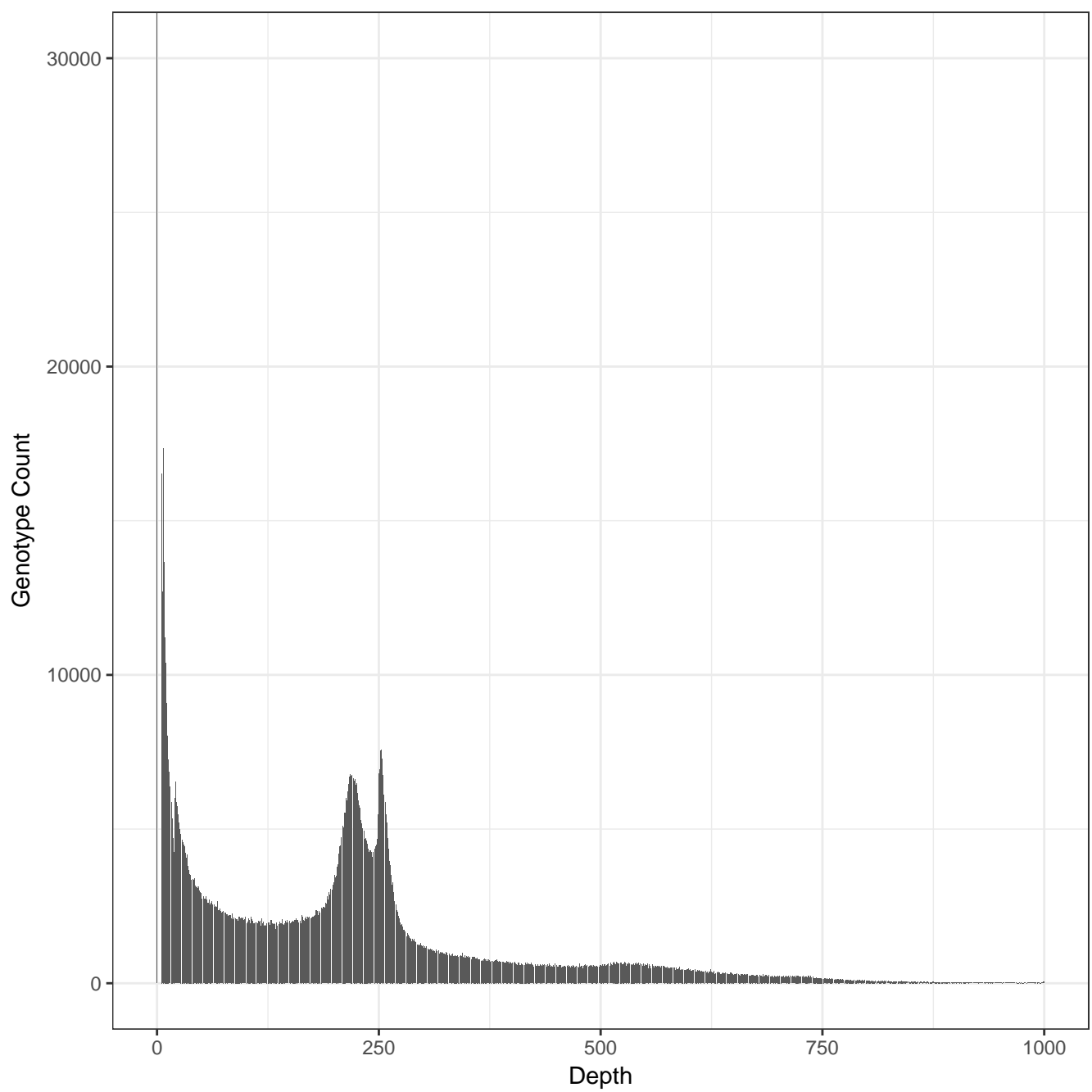

Supplement: Supplementary file 1 [file EVA-11-193-s001.pdf]
